# Supplementary material for: A new tractable method for generating human alveolar macrophage-like cells in vitro to study lung inflammatory processes and diseases
Source: mBio. 2023 Jun 8;14(4):e00834-23. doi: 10.1128/mbio.00834-23 (PMC10470505; doi:10.1128/mbio.00834-23)
Supplement: Supplemental Material — This PDF file includes the following: detailed supplemental methods; detailed supplemental references; Figures S1 to S6 and legends; Movie S1-S4 legends. [file mbio.00834-23-s0005.pdf]

mBio

Supporting Information

A new tractable method for generating Human Alveolar Macrophage Like cells in vitro to study lung inflammatory processes and diseases

Susanta Pahari[1], Eusondia Arnett[1], Jan Simper[1,2], Abul Azad[1], Israel Guerrero-Arguero[1], Chengjin Ye[1], Hao Zhang[3], Hong Cai[3], Yufeng Wang[3], Zhao Lai[4], Natalie Jarvis[1,2, ¥], Miranda Lumbreras[1, §], Diego Jose Maselli[5], Jay Peters[5], Jordi B Torrelles[1], Luis Martinez-Sobrido[1], Larry S Schlesinger[1]*

*corresponding author: Prof. Larry S. Schlesinger

Email: lschlesinger@txbiomed.org

This PDF file includes:

Detailed supplemental methods section
Detailed supplemental References
Figures and Legends S1 to S6
Movie S1-S4 Legends

Detailed supplemental methods section

Materials and methods

Collection and isolation of HAM

For isolation and culturing of HAM from BAL (1), samples obtained within 6 h were centrifuged and washed twice in PBS at 4℃, and the cell pellet was re-suspended in RPMI 1640 with Penicillin-G (Pen-G stock diluted to 1:50 in the culture medium to achieve 10,000U/mL) for adherence of HAM (Pen-G was removed after adherence). A portion of the cell suspension was subjected to cytospin, followed by staining and microscopy to evaluate the percentage of macrophages in BAL (>98%) as described below. After counting on a hemocytometer, HAM ($1x10^5$) were plated for morphometric microscopic analysis. In a parallel experiment, HAM ($5x10^5$) were lysed immediately in TRIzol reagent (Invitrogen) and samples collected for RNA-seq.

In vitro development of Alveolar Macrophage-Like (AML cells) and monocyte derived macrophages (MDM) from human PBMCs

PBMCs were isolated from healthy human donors by Ficoll-Paque cushion centrifugation. The isolated cells were cultured in sterile Teflon wells ($2x10^6$/ml) with RPMI 1640 +10% fresh autologous serum at 37°C/5% CO_2 for 6 days to allow for differentiation of monocytes into untreated MDM (2, 3) or AML cells, the latter in the presence of lung-associated surfactant components and cytokines to mimic the alveolar environment. Infasurf (calfactant) is a bovine-derived natural surfactant replacement therapeutic for preterm neonates with respiratory distress syndrome (RDS) that contains 0.7 mg hydrophobic SP-B and SP-C, phospholipids (26 mg phosphatidylcholine with 16 mg as desaturated phosphatidylcholine) and neutral lipids. To generate AMLs, Infasurf (100 μg/ml), GM-CSF (10 ng/ml), TGF-β (5 ng/ml) and IL-10 (5 ng/ml) was added on day 0, day 2, and day 4 (three doses of ALL cocktail). For some experiments, Infasurf, GM-CSF, TGF-β, and IL-10 were only added on day 0 (one dose of ALL cocktail). In other experiments, we analyzed the role of individual components of the ALL cocktail. On day 6, both control MDM and AML cells were harvested and adhered to tissue culture dishes for 2 h in RPMI 1640 with 10% fresh autologous serum, lymphocytes were washed away, and then all experiments were performed. On day 6 we typically recovered 80-90% of the total cell yield going into the Teflon wells on day 0; 10-15% of which were AML cells or MDM. Such MDM/AML cell monolayers are 99% pure and viable. We also determined the requirement of the continuous addition of ALL cocktail after differentiation to retain the phenotype of AML cells. Briefly, day 6 MDM and AML cells ($2x10^5$) were plated in a 24 well plate, non-adherent cells removed after washing 3 times with RPMI 1640 and adherent macrophages were then treated with or without ALL cocktail for 24h, 48h, and 72h. An additional experiment was performed to verify that the AML phenotype after can be retained for longer (6 more days) with ALL cocktail supplementation. At each time point cells were collected in TRIzol to quantify the expression of several genes by qRT-PCR.

Isolation of human monocytes by magnetic sorting and development of AML cells

PBMCs were isolated from healthy human donors and processed for CD14 positive monocyte isolation by negative selection using the EasySep Human Monocyte Isolation Kit (Stem cell Technologies, Cologne, Germany), according to the manufacturer's instructions. Isolated monocytes were cultured in sterile Teflon wells ($2x10^6$/ml) with RPMI 1640 + 10% fresh autologous serum at 37°C/5% CO_2 for 6 days to allow for differentiation of purified monocytes (> 90% purity) into AML cells or untreated MDM (2, 3). To generate AML cells, Infasurf (100 µg/ml), GM-CSF (10 ng/ml), TGF-β (5 ng/ml) and IL-10 (5 ng/ml) were added on day 0, day 2 and day 4. On day 6, cells were harvested and used for experiments. Such MDM/AML cells are more than 99% pure and viable. Cell lysates and supernatants were collected for real time PCR and Luminex assay, respectively, to compare differentiated AML cells from PBMCs or freshly isolated monocytes.

Annexin V and EthD-2 cell viability assay

Healthy human PBMCs were cultured for 6 days to allow for differentiation of monocytes into untreated MDM or treated AML cells. Infasurf (100 µg/ml), GM-CSF (10 ng/ml), TGF-β (5 ng/ml) and IL-10 (5 ng/ml) were added on days 0, 2, and 4 (three doses of ALL cocktail) to generate AML cells. Another group was kept as positive control to induce cell membrane breakage. AML cells were treated with 0.1 % triton X-100 for 5 min to induce cell and nuclear membrane breakage. The cells were then re-suspended in Annexin binding buffer [0.01 M HEPES (pH 7.4), 0.14 M NaCl and 2.5 mM $CaCl_2$]. APC-labeled Annexin V (5 µl/tube) was added to the all samples and incubated for 15 min in the dark at RT. After washing, Ethidium Homodimer-2 (EthD-2, 4µM) was added and incubated for another 10 min at RT. The cells were fixed with 2% paraformaldehyde (PFA). After washing with 1% binding buffer (400 µl), cells were analyzed by BD FACS symphony instrument and the data were analyzed using Flowjo software.

Cytospin analysis

Adherent macrophages were washed once with 1X with DPBS (Gibco) and treated with an enzyme-free cell dissociation buffer in Hanks' Balanced Salt Solution (Gibco, #13150016, Invitrogen). The cells were then harvested slowly with chilled RPMI 1640 and washed by centrifugation at 150 x g for 10 min. Shandon non-coated cytoslides (Thermo Scientific) were placed in a Shandon EZ single cytofunnel with white filter cards. A single cell suspension was made and 200 µL of cells ($5x10^4$) were placed into the cytofunnel and cells centrifuged at 150 x g for 5 min (Program 1). The cells in cytoslides were dried and then stained with HEMA 3 differential staining (Fisher Health Care). The slides were then washed with water and dried. They were examined with an AE2000 microscope (Motic).

Transmission electron microscopy

Cultured AML and MDM cells were washed once with 1X DPBS (Gibco) and treated with an enzyme-free cell dissociation buffer in Hanks' Balanced Salt Solution (Gibco, #13150016, Invitrogen). The cells were then harvested slowly with chilled RPMI 1640 and

washed by centrifugation at 150 x *g* for 10 min. The cell pellet was fixed with 4% formaldehyde and 1% glutaraldehyde in phosphate buffer (Invitrogen, product number: 4CF1G) overnight at 4˚C. The samples were then rinsed with 0.1M phosphate buffer. The post fixation step was performed with a Zetterqvist's buffered osmium tetroxide solution (1%) at room temperature for 30 min. The samples were then rinsed thoroughly and dehydrated gradually using 70% ethanol (1X), 95% ethanol (1X), 100% ethanol (2X), and then propylene oxide (2X) for 10 min each. Infiltration of Polybed 812 resin (Polyscience Inc.) and propylene oxide (1:1) were carried out for 30 min and to 100% resin and dried under 25 psi vacuum for 30 min. The sections were cut in 100 nm thickness using an Ultramicrotome Leica EM UC7 (Reichert-Jung, Reichert). Each grid was counterstained with uranyl acetate (5%) for 12 min and Reynold's lead citrate (80 mM lead nitrate in 164 mM sodium citrate buffer) for 5 min. Samples were imaged using a JEOL 1400 Transmission Electron Microscope (Tokyo, Japan). The images were generated in the Electron Microscopy Laboratory in the Department of Pathology and Laboratory Medicine, UT Health San Antonio.

RNA isolation, quantification and qRT-PCR

Cultured AML and MDM cells were washed once with 1X DPBS (Gibco). Monolayer cultures were treated with TRIzol and total RNA was extracted with chloroform and precipitated with isopropanol-20µg/mL Glycogen (Thermo Fisher) using the manufacturer's RNA extraction protocol (Invitrogen). The precipitated RNA pellet was washed twice with 75% ethanol and RNA reconstituted with DNase/RNase-free water. RNA was reversed-transcribed using random primers and SuperScript III Reverse Transcriptase (Invitrogen) and the condition for cDNA synthesis using reverse transcriptase was 65°C for 5 min followed by 25°C for 5 min, 50°C for 60 min, 70°C for 15 min, 4°C for infinity. The mRNA expression was evaluated by quantitative real-time RT-PCR (qRT-PCR) using TaqMan Universal PCR Master Mix (Applied Biosystems). The amplification conditions were 50°C for 10 min, 95°C for 10 min followed by 40 cycles of 95°C for 15s and 60°C for 60s in the Applied Biosystems 7500 Real-Time PCR System. Predesigned TaqMan human primers with best coverage and most citations (Thermo Fisher Scientific) were selected. Relative expression was calculated by the ΔΔ threshold cycle (ΔΔCT) method using β-actin (ACTB) as the housekeeping gene. Expression levels of basal mRNA in AML and MDM cells were normalized to ACTB.

Multi-color flow cytometry

Adherent AML and MDM cells were washed once with 1% DPBS (Gibco) and treated with enzyme-free cell dissociation buffer in Hanks' Balanced Salt Solution (Gibco, #13150016, Invitrogen). Single cell suspensions were made, placed in Polypropylene FACS tubes (Becton Dickinson) and incubated with human TruStain FcX™ (Fc receptor blocking solution # 422302, Biolegend) at room temperature for 5-10 min. This was followed by incubation of cells with fluorochrome tagged antibodies (BD Biosciences, San Jose, CA and Biolegend, San Diego, CA): anti-CD45 (clone D058-1283), anti-CD64 (clone 10.1), anti-CD206 (clone 19.2), anti-CD11c (clone 3.9), anti-MerTK (clone 590H11G1E3), anti-CD163 (clone GHI/61), anti-CD170 (clone 1A5/CD170), anti-CD11b

(clone ICRF44 or M1/70), anti-CD36 (clone 5-271), anti-MARCO (clone PLK-1) and anti-HLA-DR (clone L243) along with their respective isotype matched control antibodies at 4°C for 30 min. Samples were analyzed using a multi-color BD FACS symphony instrument (Becton Dickinson) and compensation, analysis and visualization of the data were performed using Flowjo software (Williamson Way). Isotype and "Fluorescence minus one" controls were used, when necessary, to set up gates. The gating strategy used to analyze the macrophages is represented in **Fig. S5**.

Multicolor confocal microscopy

Single cell suspensions of AML cells and MDM were made. The cells ($1x10^5$) were placed in FACS tubes (BD), washed with cell staining buffer (Biolegend) and incubated with human TruStain FcX™ (Fc receptor blocking solution, #422302, Biolegend) at room temperature for 30 min. This was followed by incubation of cells with fluorochrome tagged antibodies (BD Biosciences and Biolegend) in several panels: [(Panel 1: anti-CD206 FITC (clone 19.2), anti-CD11c PE (clone 3.9), CD200R APC (clone OX-108)], [Panel 2: anti-CD170 APC (clone 1A5/CD170), anti-CD64 PE (clone 10.1), CD68 Alexa Fluor 488 (clone Y1/82A)], [(Panel 3: anti-CD163 PE (clone GHI/61), anti-CD11b Alexa Fluor 488 (clone ICRF44 or M1/70), anti-CD36 APC (clone 5-271)] and [Panel 4: anti-MerTK PE (clone 590H11G1E3) anti-MARCO (clone PLK-1)] along with their respective isotype matched control antibodies at 4°C for 45 min. Cells were washed with cell staining buffer with centrifugation at 250 x g for 10 min at RT. The stained cells (5×10^4/ 200 µL) were placed in the cytofunnel. Centrifugation was performed in the Shandon Cytospin 4 at 150 x g for 5 min (Program 1). The cells were then fixed with 2% paraformaldehyde for 3 min. The slides were washed 3 times with 1X DPBS (Gibco) and mounted on coverslips with prolong gold along with DAPI (Thermo Fisher Scientific). The stained slides were visualized with a Ziess LSM 800 confocal microscope (20X, 63X magnification). Mean fluorescence intensity (MFI) and percentage of positive cells were counted by ZEN 2.3 blue edition (Zeiss) and ImageJ software (NIH, Bethesda, MA).

Bead cell association study

AML and MDM cells were placed in chamber slides (Corning) for adherence and washed to remove non-adherent cells. Non-opsonized FluoSpheres™ Sulfate Microspheres, 1.0 µm, yellow-green fluorescent F8852-beads (Invitrogen) were added (MOI 10) and incubated at 37°C/5% CO_2 incubator for 45 min in RHH (RPMI 1640 + 10 mM HEPES + 0.4% human serum albumin). Extracellular beads were removed after washing 3 times with RHH media. The cells were then incubated with DAPI (0.4 µg/mL) for 10 min and next fixed with 2% paraformaldehyde for 3 min. The slides were washed 3 times with 1X DPBS (Gibco) and mounted on coverslips with ProLong Gold Antifade Mountant with DAPI (Thermo Fisher Scientific). Stained slides were visualized with a Ziess LSM 800 confocal microscope (63X magnification). The cells were counted based on DAPI staining. The number of fluorescent beads was also counted and shown as beads/macrophage.

RNA-Seq and analyses

Freshy isolated HAM and 2h adherent MDM and AML cells were washed once with 1X DPBS (Gibco), lysed in TRIzol (Invitrogen) and RNA was isolated using Direct-zol RNA Miniprep kit, R2052 (Zymo Research) as per the manufacturer's instructions. Isolated RNA was quantified using the Qubit 4 Fluorimeter (Invitrogen). RNA quality was assessed with the 4200 TapeStation System (Agilent). Samples with an RNA integrity number (RIN) higher than 7 were used for RNA-seq. RNA-seq libraries were prepared from 300ng of total RNA using the mRNA-seq library preparation and 50bp single read sequencing with approximately 25-30M reads per sample in the NEB Next RNA Ultra Kit (Qiagen, Redwood City, CA) with poly (A) enrichment. RNA sequencing was carried out at the Genome Sequencing Facility (GSF) at UTHealth San Antonio using the HiSeq 3000 platform (Illumina).
Raw sequencing reads were preprocessed using Trim Galore! to trim the adapters and low-quality sequences (4). Trimmed reads were mapped to the human hg38 reference genome using HISAT2(5). Read counts for each sample were obtained using feature Counts (6). Differential gene expression analysis was carried out using DESeq2 (7). The Benjamini-Hochberg procedure was used to control the false discovery rate (FDR), and the shrunken log2 fold changes (LFCs) were calculated using the adaptive shrinkage (ash) estimator with an Empirical Bayes approach. Genes with FDR-adjusted p-value <0.05 and LFC of more than 1 or less than -1 were considered to be differentially expressed. Gene Set Enrichment Analysis (GSEA) was carried out to identify over-represented gene ontology (GO) terms and biological pathways (8). Pathway and network analyses were performed using Ingenuity Pathway Analysis (IPA) (Qiagen). Functional protein association networks were analyzed using StringApp (9, 10) and network visualization was generated using Cytoscape version 3.8.2 (11). Heatmaps of specific genes were generated using the pheatmap package in R.

Luminex-multiplex analysis

Luminex assays were used to measure cellular release of cytokines, chemokines and other secretory factors in the culture supernatant. The analytes IL-1β, IL-2, IL-4, IL-6, IL-8, IL-10, IL-12 p70, IL-13, IL-18, TNFα, IFN-α, IFN-γ, CCL5/RANTES, CCL8, CCL18, CCL22, CD163, GM-CSF, ICAM-1, M-CSF, MIF, MMP-1, MMP-7, MMP-9, MMP-12 and RAGE were measured by Luminex Human Discovery Assay (25-Plex, code 9VeEpjBu) LXSAHM-25 kit (R&D Systems, Inc.). Briefly, 2 x 10^6/mL of total PBMCs and MACS purified monocytes were cultured as untreated (UT-MDM) or All Cocktail treated AML (ALL-AML). On day 6, culture supernatants were collected and cells were processed for experiments. Supernatants were measured for the presence of the released factors following the manufacture's protocol by the Luminex® 100/200™ System (Luminex Corporation). The data were analyzed by Belysa™ Immunoassay Curve Fitting Software (Millipore Sigma).

Western blot analysis

Adherent monolayers of 6-day old AML and MDM cells ($2x10^6$) were washed once with 1X DPBS (Gibco) and dissociated using Hanks' Balanced Salt Solution (Gibco, Invitrogen). The cells were lysed with NE-PER Nuclear and Cytoplasmic Extraction kit (Thermo Scientific, R2052) according to the manufacturer's protocol. Protein concentration in the nuclear samples was measured by BCA assay (Thermo Scientific). The nuclear proteins (30 µg/sample) were denatured and reduced proteins were resolved on 10 or 12 % SDS-PAGE and transferred to nitrocellulose membranes. The membranes were then blocked with 5% skim milk for 2h and incubated overnight with the primary antibody for PPAR-γ (Cell Signaling Technology; 1:1000), PU.1 (Cell Signaling Technology; 1:1000), H3K4me1 (Thermo Scientific; 1:1500) and H3K4me3 (Thermo Scientific; 1:1000) in 5% BSA with TBST (1X Tris-Buffered Saline, 0.1% Tween 20) at 4°C. The membranes were washed and incubated with anti-rabbit IgG, HRP-linked antibody (1:2500-1:4000 dilutions) diluted in TBST for 30 min. The membranes were then washed and developed using clarity ECL reagent on a UVP chemstudio 815 system (AnalytikJena). The membranes were next stripped, then blocked with 5% skim milk/ BSA and probed for β-actin (anti-rabbit HRP conjugated, CST, 1:2000) or Histone H3 (Thermo Scientific; 1:1000) levels. Protein band intensities were determined with VisionWorks, for each sample background values were subtracted and then values were normalized to the β-actin loading control.

Extracellular Flux Analysis (Agilent Seahorse)

Real-time cell metabolism of AML cells and MDM was determined by measuring oxygen consumption rate (OCR, pmol/min) and extracellular acidification rate (ECAR, mPh/min) using a Seahorse XF Extracellular Flux Analyzer XFe96 (Agilent Technologies), according to the manufacturer's instructions. Briefly, AML cells and MDM ($5x10^4$/well) were adhered in 96 well Seahorse plates for 1h. The cells were then washed and replenished with XF DMEM Seahorse media supplemented with 25 mM D-Glucose, 1 mM Sodium pyruvate, and 2 mM L-glutamine. After incubation in a non-CO_2 incubator at 37˚C for 1h, basal levels of OCR and ECAR were measured. Mito stress assay was performed after sequential addition of 5 µM oligomycin (ATP synthesis inhibitor), 4 µM carbonyl cyanide 4-(trifluoromethoxy) phenylhydrazone (FCCP, uncoupling agent) and 2 µM rotenone and antimycin A (inhibitors of complex I and III of the respiratory chain, respectively). For glycolysis stress analysis, AML and MDM cells were injected with 2 µM rotenone and 2 µM antimycin A followed by 100 mM 2-deoxyglucose (2-DG) to determine glycolytic rate. All of these components have specific roles as follows. Oligomycin (O), an inhibitor of ATP synthase (complex V) which decreases electron flow through the ETC, was injected to verify the basal OCR measurement impacts on mitochondrial respiration or OCR. A decrease in OCR is directly related to cellular ATP production. Carbonyl cyanide-4 (trifluoromethoxy) phenylhydrazone (FCCP) is a well-known uncoupler which blocks the proton gradient and interrupts the mitochondrial membrane potential resulting in the continuous electron flow through the ETC and maximal level of oxygen consumption by complex IV. The spare respiratory capacity (SRC) was calculated by measuring the

difference between FCCP-induced maximal respiration and basal respiration. The third injection uses a combination of rotenone (complex I inhibitor) and antimycin A (complex III inhibitor), which blocks mitochondrial respiration, enabling measurement of non-mitochondrial respiration in the cells. In the ECAR glycolytic rate assay, glycolytic pathway inhibitor 2-deoxy-glucose (2-DG) is used to inhibit glycolysis through competitive binding to glucose hexokinase.

Mitosox assay and cellular ROS detection

Mitochondrial reactive oxygen species (ROS) production in AML cells and MDM was determined by staining with 5μM of Mitosox (Thermo fisher Scientific, Inc.) for 30 min for confocal or flow cytometry analysis. Oxidation of H2DCF by intracellular non-mitochondrial ROS levels in living AML and MDM cells was measured by confocal microscopy and flow cytometry using a cytosolic ROS detection assay kit according to the manufacturer's instruction (K936, BioVision Inc, Milpitas). The flow cytometry data were analyzed using FlowJo (FlowJo LLC-BD) and further analyses were conducted by GraphPad Prism 9 (Graphpad).

EPR assay

Electron paramagnetic resonance (EPR)-based ROS detection for mtO_2 was performed using Mito-Tempo-H. Briefly, AML cells and MDM were incubated in EPR buffer (10 mM D-Glucose, 20 mM HEPES, 0.7 mM Na_2HPO_4, 25 mM $NaHCO_3$, 1.3 mM NaH_2PO_4, 4.5 mM KCl, 0.5 mM MgCl, 25 µM deferoxamine and 5 µM DETC) including 100 µM Mito-Tempo-H, for ~60 min at 37 °C. 2 µM rotenone and 2 µM antimycin A mix were added to inhibitor complex I and III of the respiratory chain in mitochondria. The samples were then flash frozen in -80˚C for 1h to lyse cells and cell supernatants were collected after centrifugation (250 x *g* for 5 min at RT). The EPR spectra were measured on the Bruker EMXnano ESR system (Bruker Corporation). The 2D spectra were combined, baseline corrected, and assessed using spinfit and GraphPad software.

Phagocytosis assay for *M.tb*

HAM, AML or MDM monolayers ($1.5x10^5$ cells/well) were seeded on coverslips and mCherry-*M.tb* $H_{37}R_v$ added at an MOI of 5 in RHH (RPMI 1640 + 10 mM HEPES + 0.4% human serum albumin). After 2h incubation, the monolayers were washed to remove extracellular bacteria, fixed with 4% paraformaldehyde for 10 min (non-permeabilized), washed and blocked overnight with 5% BSA and 10% FBS in DPBS. Monolayers were then incubated with either rabbit polyclonal anti-*M.tb* whole cell lysate antibody (BEI Resources, NIAID, NR-13819) or an IgG rabbit isotype control antibody (1:1000 for 2h in blocking buffer). After washing, monolayers were incubated with an AlexaFluor 488 donkey anti-rabbit secondary antibody (Invitrogen; 1:500 incubate for 1h). The coverslips were then washed with DPBS and mounted on glass slides with ProLong™ Gold Antifade Mountant with DAPI. The imaging was performed on a Zeiss LSM 800 microscope (Zeiss, NY). Phagocytosed/internalized *M.tb* demonstrated red fluorescence and surface-associated *M.tb* demonstrated green/yellow fluorescence. Approximately 100

macrophages were examined for differential counting of *M.tb* bacilli. Data are represented as the number of *M.tb* per cell and percent positive macrophages with ≥ 1 bacterium.

Macrophage infection with *M.tb*

Single cell suspensions of *M.tb* $H_{37}R_v$ were prepared as described (12, 13). *M.tb* $H_{37}R_v$ was used to infect macrophage monolayers in a 96-well plate at MOI 2. After 2h incubation, the infected monolayers were washed 3x, replaced with fresh RPMI+2% autologous serum and incubated for 2, 24, 48 and 72h. CFU counting was done to measure uptake at the 2h time point. At each time point post-infection (2, 24, 48 and 72h), *M.tb* intracellular growth was assessed in macrophage lysates. Dilutions of lysates were plated on 7H11 agar media to allow for bacterial growth, followed by bacterial counting for colony-forming unit (CFU) assessment after 3 and 4 weeks.

Generation of rSARS-CoV-2 expressing reporter genes

Recombinant SARS-CoV-2 expressing mCherry and nanoluciferase (Nluc) reporter genes (rSARS-CoV-2/mCherry-Nluc) was rescued as previously described (14). Briefly, confluent monolayers of Vero E6 cells ($1x10^6$ cells/well, 6 well plates, four wells) were transfected using Lipofectamine 2000 (LPF2000; Thermo Fisher) with pBeloBAC11-SARS-CoV-2/WT (4 mg/well) or pBeloBAC11-SARS-CoV-2/mCherry-Nluc. The transfection medium was replenished after 12-14h with post-infection medium (DMEM with 2% FBS). After 24h, cells were transfer into T75 flasks to scale up culture. 72 h later, virus-containing culture supernatants were collected and stored at -80°C. Viability of active rSARS-CoV-2/mCherry-Nluc virus was confirmed by infection of fresh monolayers of Vero E6 cells ($1x10^6$ cells/well) in 6 well plates by assessing expression of mCherry as well as Nluc. After confirming viral rescues, viruses were passaged to generate viable viral stock. The samples were then flash frozen in -80˚C overnight to lyse rSARS-CoV-2-mCherry-Nluc infected Vero E6 cells and cell supernatants were collected after centrifugation (250 x g for 5 min at RT). Vero E6 cell contamination in viral suspension was further minimized by passing through 0.22 µM filters. Viral titers were determined by standard plaque assay (plaque forming units, PFU/mL) and quantified by immunostaining with a SARS-CoV N protein cross-reactive antibody (1C7C7) in Vero E6 cells (10^6 cells/well; 6 well plate format).

Macrophage infection with rSARS-CoV-2/mCherry-Nluc

African green monkey kidney epithelial cells (Vero E6, CRL-1586) ($2x10^4$ /well, 96 well plate format, quadra wells) were cultured with DMEM-10% FBS (heat-inactivated Fetal Bovine Serum)/1% Penicillin/Streptomycin/L-glutamine solution the day before each experiment to allow for adherence and confluence. Freshly isolated HAM ($5x10^4$), AML cells and MDM monolayers were washed, and along with Vero E6 cells, were infected with rSARS-CoV-2-mCherry-Nluc virus at MOI of 1 or 10 PFU/cells for 2h at 37˚C in a $C0_2$ incubator. After viral absorption, infectious media was removed, washed carefully with RPMI-2% autologous serum for HAM, AML cells and MDM, and RPMI-2% FBS for Vero E6 cells, and repleted with fresh post-infection media.

Cytation 5 live cell imaging assay

Freshly obtained HAM, AML, MDM and confluent monolayers of Vero E6 cells ($5x10^4$ cells/well, 96 well plates, quadra wells) were infected with rSARS-CoV-2/mCherry-Nluc virus at MOI 1 and 10 ($5x10^4$ and $5x10^5$) for 2h at 37°C in a $C0_2$ incubator. Following washing and media repletion, live cell imaging was performed using Citation 5 paired with BioSpa (Biotek/Agilent) under 20X magnification. The images were acquired every 12h for 84h. Data analysis was performed with Gen5 software (Biotek/Agilent) by calculating mCherry mean fluorescence intensity. mCherry background signal was determined in uninfected control wells and subtracted from mCherry MFI in each well by Gen5 data analysis software. Cells were counted after 120h by counter staining with DAPI and imaging with the Cytation 5 live imager. The presence of SARS-CoV-2-mCherry was monitored in each cell type in time lapse videos (0/4-84h time period) using Gen5 software.

Detailed supplemental References

1. Gaynor CD, McCormack FX, Voelker DR, McGowan SE, Schlesinger LS. 1995. Pulmonary surfactant protein A mediates enhanced phagocytosis of Mycobacterium tuberculosis by a direct interaction with human macrophages. J Immunol 155:5343-51.
2. Schlesinger LS, Horwitz MA. 1990. Phagocytosis of leprosy bacilli is mediated by complement receptors CR1 and CR3 on human monocytes and complement component C3 in serum. J Clin Invest 85:1304-14.
3. Schlesinger LS. 1993. Macrophage phagocytosis of virulent but not attenuated strains of Mycobacterium tuberculosis is mediated by mannose receptors in addition to complement receptors. J Immunol 150:2920-30.
4. Krueger F. 2012. Trim Galore! https://www.bioinformatics.babraham.ac.uk/projects/trim_galore/.
5. Kim D, Langmead B, Salzberg SL. 2015. HISAT: a fast spliced aligner with low memory requirements. Nat Methods 12:357-60.
6. Liao Y, Smyth GK, Shi W. 2014. featureCounts: an efficient general purpose program for assigning sequence reads to genomic features. Bioinformatics 30:923-30.
7. Love MI, Huber W, Anders S. 2014. Moderated estimation of fold change and dispersion for RNA-seq data with DESeq2. Genome Biol 15:550.
8. Subramanian A, Tamayo P, Mootha VK, Mukherjee S, Ebert BL, Gillette MA, Paulovich A, Pomeroy SL, Golub TR, Lander ES, Mesirov JP. 2005. Gene set enrichment analysis: a knowledge-based approach for interpreting genome-wide expression profiles. Proc Natl Acad Sci U S A 102:15545-50.
9. Szklarczyk D, Gable AL, Lyon D, Junge A, Wyder S, Huerta-Cepas J, Simonovic M, Doncheva NT, Morris JH, Bork P, Jensen LJ, Mering CV. 2019. STRING v11: protein-protein association networks with increased coverage, supporting functional discovery in genome-wide experimental datasets. Nucleic Acids Res 47:D607-D613.

10. Doncheva NT, Morris JH, Gorodkin J, Jensen LJ. 2019. Cytoscape StringApp: Network Analysis and Visualization of Proteomics Data. J Proteome Res 18:623-632.
11. Shannon P, Markiel A, Ozier O, Baliga NS, Wang JT, Ramage D, Amin N, Schwikowski B, Ideker T. 2003. Cytoscape: a software environment for integrated models of biomolecular interaction networks. Genome Res 13:2498-504.
12. Schlesinger LS, Bellingerkawahara CG, Payne NR, Horwitz MA. 1990. Phagocytosis of Mycobacterium-Tuberculosis Is Mediated by Human Monocyte Complement Receptors and Complement Component-C3. Journal of Immunology 144:2771-2780.
13. Salunke SB, Azad AK, Kapuriya NP, Balada-Llasat JM, Pancholi P, Schlesinger LS, Chen CS. 2015. Design and synthesis of novel anti-tuberculosis agents from the celecoxib pharmacophore. Bioorg Med Chem 23:1935-43.
14. Ye C, Chiem K, Park JG, Oladunni F, Platt RN, 2nd, Anderson T, Almazan F, de la Torre JC, Martinez-Sobrido L. 2020. Rescue of SARS-CoV-2 from a Single Bacterial Artificial Chromosome. mBio 11.

Supplemental Figures

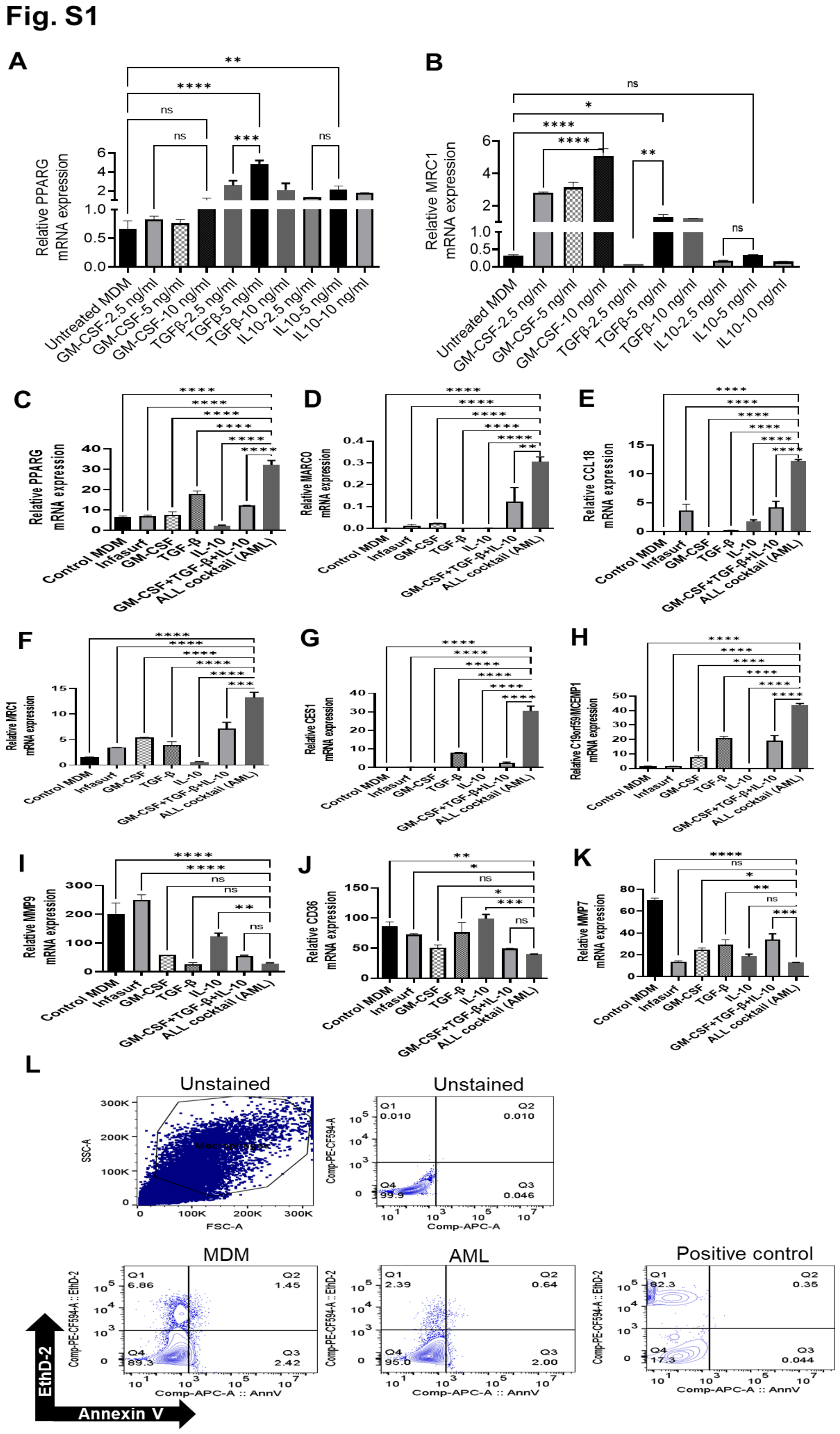

Fig. S1. Selection of the optimal dose of GM-CSF, TGF-β and IL-10 and role of lung-associated components individually and in combination in generating AML cells. PBMCs from healthy human donors were exposed to lung-associated component treatment [cytokines GM-CSF (2.5, 5, and 10 ng/mL), TGF-β (2.5, 5, and 10 ng/mL) and IL-10 (2.5, 5, and 10 ng/mL)] for 6 days on alternative days or left untreated (MDM). The optimum concentration was selected by measuring gene expression of (A) PPARG and (B) MRC1 by qRT-PCR. Gene expression was normalized to Actin. Representative bar diagram showing the relative mRNA expression expressed as mean ± SD (n=2 donors). PBMCs from healthy human donors were exposed to lung-associated component treatment [surfactant (Infasurf: 100 µg/mL) and cytokines (GM-CSF: 10 ng/mL, TGF-β: 5 ng/mL, IL-10: 5 ng/mL) alone or all together (ALL treated)] for 6 days on alternative days (Day 0, 2, 4), or left untreated (MDM). qRT-PCR data demonstrated significant increases in expression of (C) PPARG, (D) MARCO, (E) CCL18, (F) MRC1, (G) CES1 and (H) MCEMP1, and decreases in (I) MMP9, (J) CD36 and (K) MMP7 in the ALL cocktail group compared to treatment with each component alone. The data were normalized to the actin control. Data are expressed as mean ± SD (n=2). Data are expressed as mean ± SD and analyzed with one-way ANOVA * $p \leq 0.05$, ** $p \leq 0.01$, *** $p \leq 0.001$, **** $p \leq 0.0001$. (n=2). (L) ALL cocktail treatment does not affect the viability of AML cells. PBMCs were exposed to Infasurf (100 µg/mL) and cytokines [(GM-CSF: 10 ng/mL, TGF-β: 5 ng/mL, IL-10: 5 ng/mL) (ALL cocktail treated)] for 6 days on alternative days (Day 0, 2, 4), or left untreated (MDM). AML cells were untreated or treated with 0.1 % triton X-100 for 5 min to induce cell and nuclear membrane breakage (positive control). Cells were then stained with Annexin V (ANXA5/annexin V-APC) followed by Ethidium Homodimer-2 (EthD-2, 4µM) to assess for cell viability. The numbers in the insets indicate the percentage of Annexin V and EthD-2-positive cells. Representative data from 3 donors.

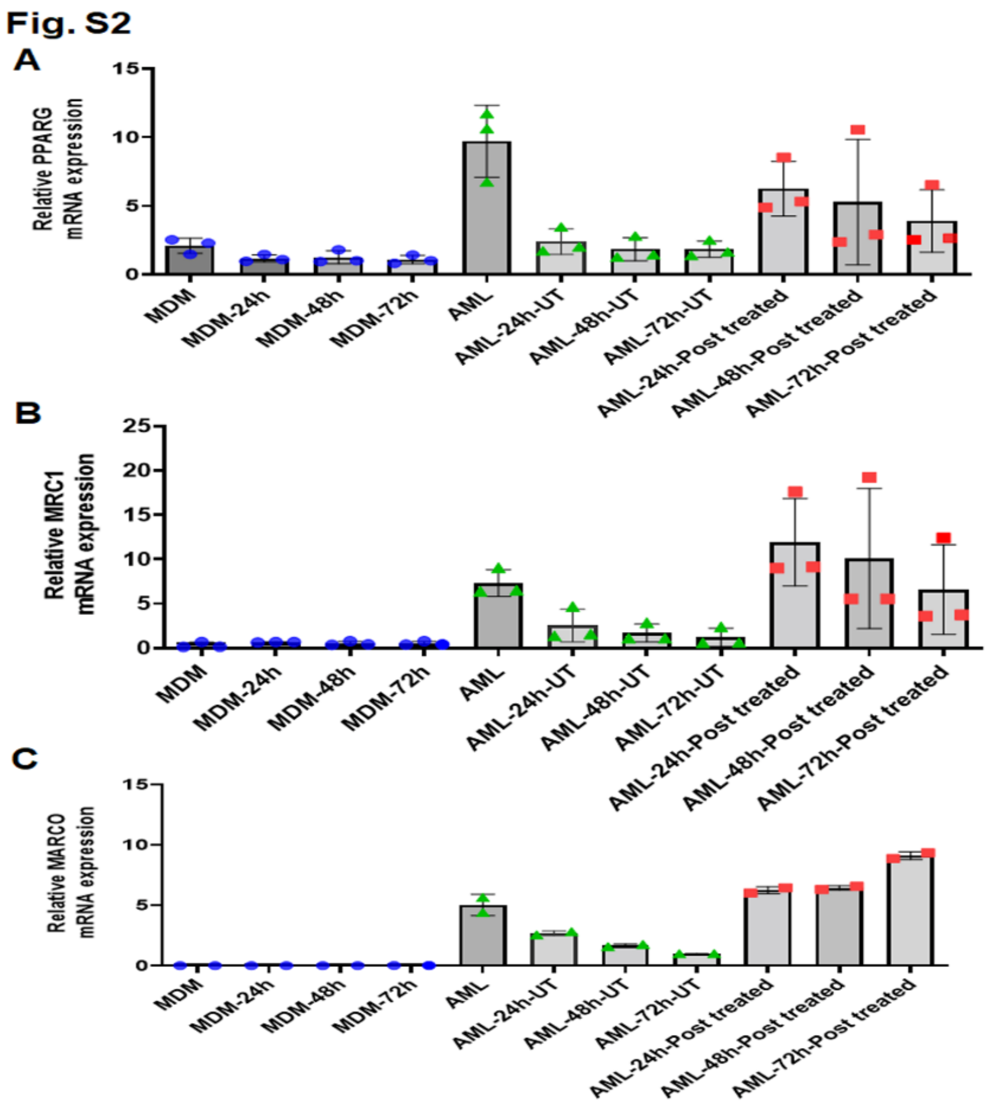

Figure S2. **Continuous supplementation of the lung-associated components is required to retain the HAM phenotype of AML cells.** PBMCs were exposed to lung-associated components treatment [Infasurf (100 µg/mL) and cytokines (GM-CSF: 10 ng/mL, TGF-β: 5 ng/mL, IL-10: 5 ng/mL)] for 6 days on alternative days, or left untreated (MDM). The adherent macrophages were then treated with or without ALL of lung-associated components [Infasurf (100 µg/mL) and cytokines (GM-CSF: 10 ng/mL, TGF-β: 5 ng/mL, IL-10: 5 ng/mL)] and incubated for 24, 48, and 72h. At each time point cells were collected to quantify the expression of (A) PPARG, (B) MRC1, and (C) MARCO by qRT-PCR. Blue circle dots show individual donors of the MDM control for 0h (day 6), 24h, 48h or 72h (without any treatment). Green triangles indicate individual donors of the AML cells treated with 3 doses of cocktail up to day 6 and cultured for 0h (day 6), 24h, 48h or 72h without additional treatment. Red squares indicate individual donors of AML cells where additional treatment supplementation (Post treated) after adherence can extend the HAM phenotype. n=3, Mean ± SEM.

Fig. S3

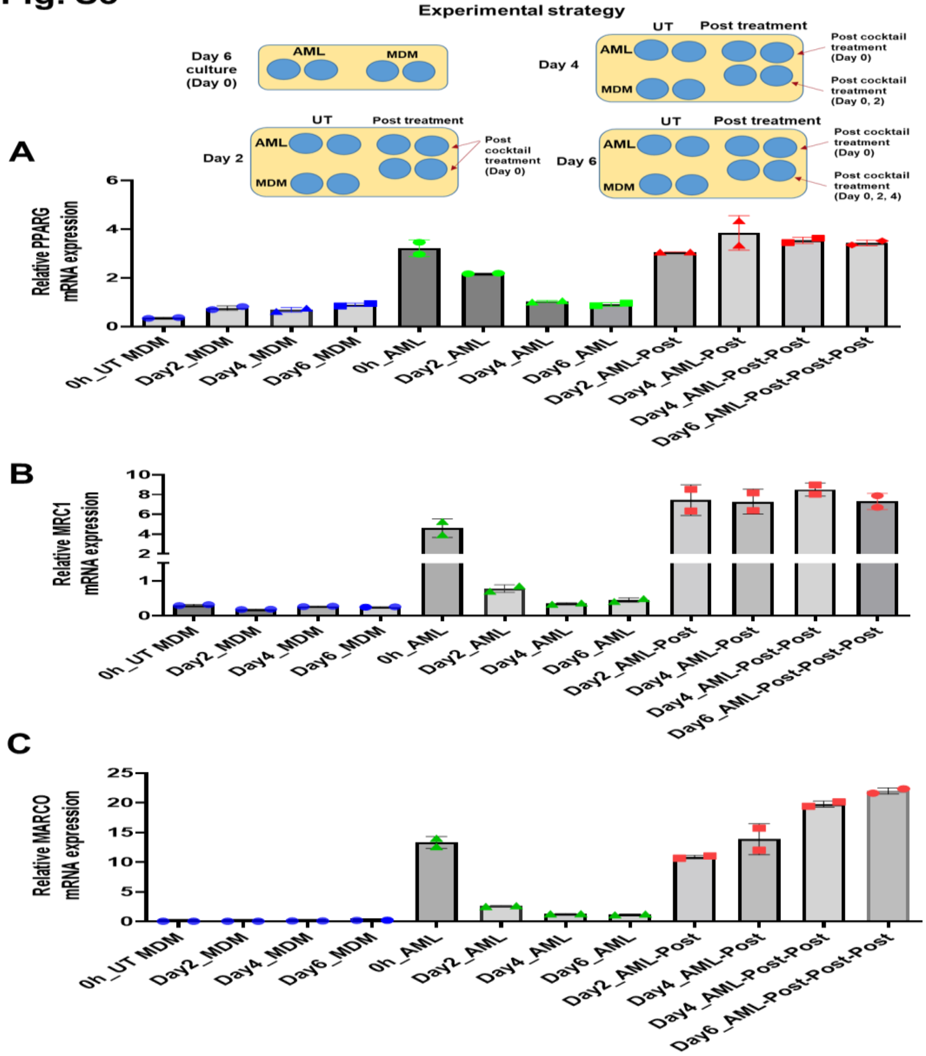

Figure S3. **Continuous supplementation of the lung-associated components retains the HAM phenotype of AML cells for longer duration.** Monocytes were purified from human PBMCs by magnetic sorting and cultured with lung-associated components [Infasurf (100 µg/mL) and cytokines (GM-CSF: 10 ng/mL, TGF-β: 5 ng/mL, IL-10: 5 ng/mL)] for 6 days on alternative days, or left untreated (MDM). On day 6, the differentiated cells were plated ($5x10^5$/ well) in a 24 well plate. Adherent cells were then treated with or without ALL cocktail (post treatment) on days 2, 4 and 6 (see illustration). At each time point cells were collected to quantify the expression of (A) PPARG, (B) MRC1, and (C) MARCO by qRT-PCR. Blue circle dots show individual donors of the MDM control for day 0 (day 6 of culture), 2, 4 or 6 (without any treatment). Green triangles indicate individual donors of the AML cells treated with 3 doses of cocktail up to day 6 differentiated culture and then cultured for day 0, 2, 4, or 6 without additional cocktail treatment. Red squares indicate individual donors of AML cells treated with 3 doses of ALL cocktail up to day 6 differentiated culture and then cultured for day 0, 2, 4 or 6 with additional treatment supplementation (Post treated) on day 0, 2 and 4. n=2, Mean ± SEM.

Fig. S4

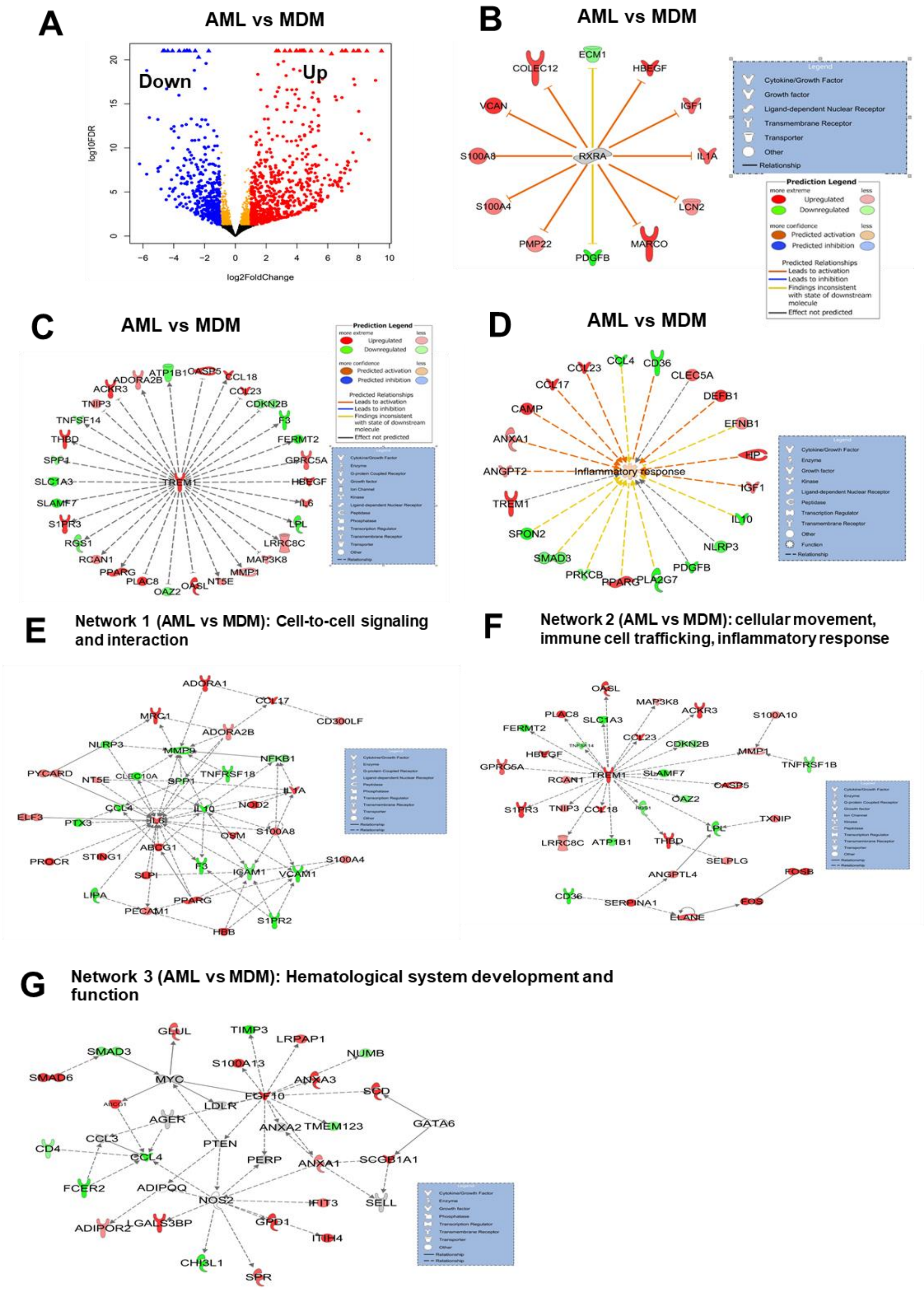

Fig. S4. AML cells and MDM share transcriptional signatures and related pathways. (A) Volcano plot demonstrates the comparison between the AML and MDM transcriptome. AML cells and MDM are more dissimilar than AML cells and HAM: out

of 14,097 expressed genes, 744 are up-regulated ≥2-fold with FDR adjusted p-value < 0.05 (red), and 438 are down-regulated ≥2-fold with FDR adjusted p-value < 0.05 (blue) in MDM. (B-D) IPA analysis identified several pathways containing genes that were significantly up-regulated in AML cells relative to MDM with similar expression in AML cells and HAM. They include involvement of (B) RXRA transcription factor with upregulation of MARCO, COLEC12, HBEGF, IGF1, S100A4 and VCAN, (C) TREM1 and (D) Inflammatory response network with involvement of PPARG and down regulation of CD36. (E-G) IPA network analysis of genes that were differentially expressed in AML cells compared to MDM identifies (E) network 1 (immune cell trafficking, cellular movement, cell-to-cell signaling and interaction), (F) network 2 (cellular movement, immune cell trafficking, inflammatory response) and (G) network 3 (immune cell trafficking, cellular movement, hematological system development and function).

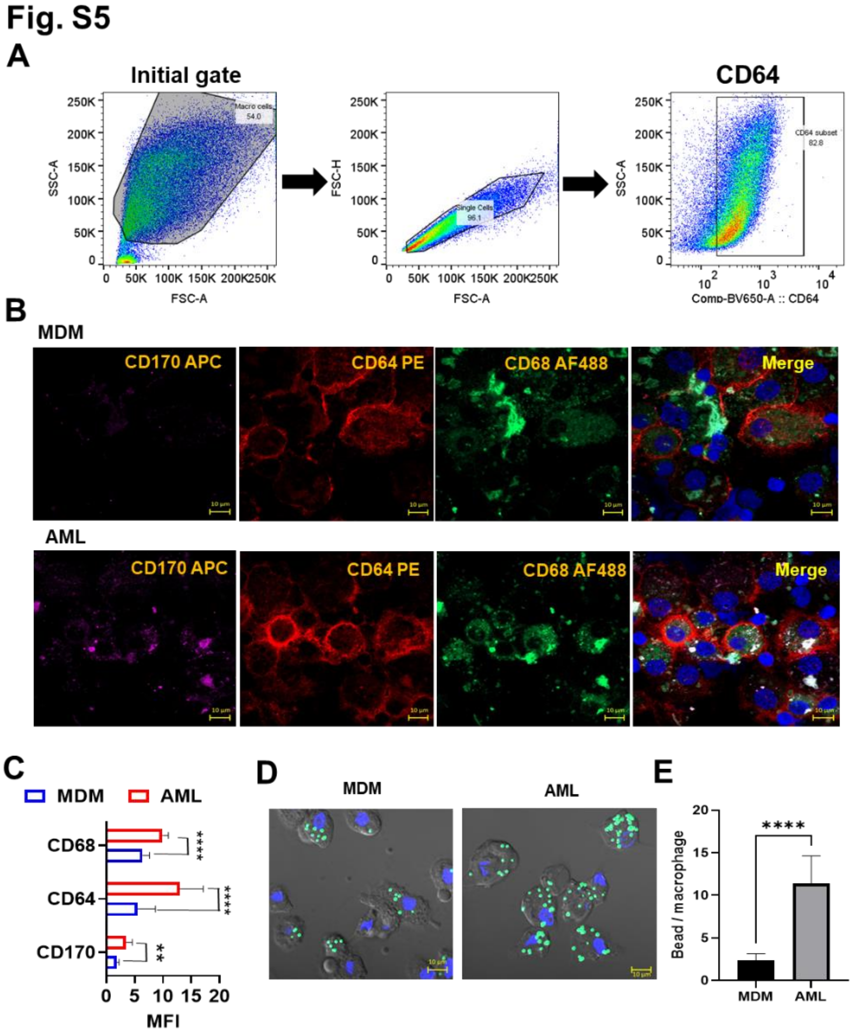

Fig. S5. Flow cytometry gating strategy for Figure 6 A-J, confocal and cell association assays. (A) FSC vs SSC was used as the initial gate, then FSC vs FSC to gate singlets. The population was then gated on CD64 positive cells (macrophages) for subsequent analysis. (B) Cells were immunostained with the indicated antibodies and DAPI for nucleus (blue),

then imaged with confocal microscopy. AML cells had higher CD170, CD68 and CD64 expression than MDM. Scale bar: 10µm and 63X magnification. (C) Confocal data were quantified by mean fluorescence intensity (MFI) and are represented as a bar graph. Representative data showing the expression of indicated proteins of 3 different donors. Data are expressed as mean ± SD and analyzed by Unpaired Student's 't' test ** p≤0.01, ****p≤0.0001. (D) Cell association study using unopsonized green fluorescent beads comparing AML cells and MDM, nuclei were stained with DAPI (blue). Scale bar: 10µm and 63X magnification. (E) Quantification of the number of beads per macrophage. Representative experiment is shown of n=2, Mean ± SD, **** p < 0.0001.

Fig. S6

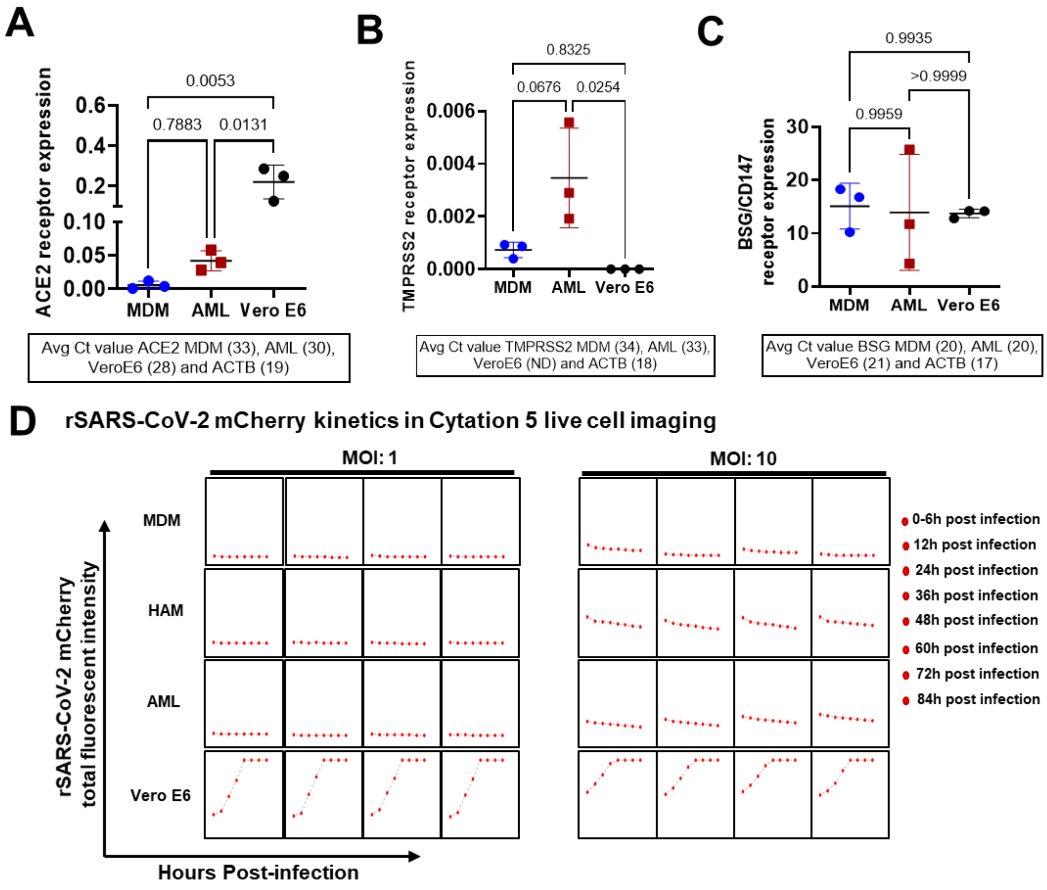

Fig. S6. AML cells express ACE2, TMPRSS2, and BSG/CD147 and have increased uptake and persistence of SARS-CoV-2 monitored by Cytation 5 live cell imaging. (A-C) ACE2, TMPRSS2 and BSG/CD147 receptor expression was measured in MDM, AML cells and Vero E6 cells by qRT-PCR. n = 3 human donors. (D) mCherry fluorescent SARS-CoV-2 in individual wells was monitored for uptake and persistence over time by the Cytation 5 live cell imaging system (MDM, HAM, AML cells and Vero E6 cells). Representative mini-graphs (of n=4) demonstrate total mCherry fluorescence units (Y-axis) versus post infection time (X-axis). Shown up to 84h post infection.

Movie S1. Video of SARS-CoV-2 mCherry viral infection in MDM using Cytation 5 live cell imaging 0/4-84h post-infection. Persistence of SARS-CoV-2-mCherry virus over time was monitored by Cytation 5 live cell imaging. The time-lapse video shows mCherry (red) SARS-CoV-2.

Movies_MPEG4 files\MDM_SARS-CoV-2_MOI 1.mp4

Movie S2. Video of SARS-CoV-2 mCherry viral infection in AML cells using Cytation 5 live cell imaging 0/4-84h post-infection. Persistence of SARS-CoV-2-mCherry virus over time was monitored by Cytation 5 live cell imaging. The time-lapse video shows mCherry (red) SARS-CoV-2.

Movies_MPEG4 files\AML_SARS-CoV-2_MOI 1.mp4

Movie S3. Video of SARS-CoV-2 mCherry viral infection in HAM using Cytation 5 live cell imaging 0/4-84h post-infection. Persistence of SARS-CoV-2-mCherry virus over time was monitored by Cytation 5 live cell imaging. The time-lapse video shows mCherry (red) SARS-CoV-2.

Movies_MPEG4 files\HAM_SARS-CoV-2_MOI 1.mp4

Movie S4. Video of SARS-CoV-2 mCherry viral infection in Vero E6 cells using Cytation 5 live cell imaging 0/4-84h post-infection. Persistence of SARS-CoV-2-mCherry virus over time was monitored by Cytation 5 live cell imaging. The time-lapse video shows mCherry (red) SARS-CoV-2.

Movies_MPEG4 files\VeroE6_SARS-CoV-2_MOI 1.mp4
